# Inhibition of Glycogen Synthase Kinase 3β Ameliorates D-GalN/LPS-Induced Liver Injury by Reducing Endoplasmic Reticulum Stress-Triggered Apoptosis

**DOI:** 10.1371/journal.pone.0045202

**Published:** 2012-09-28

**Authors:** Liyan Chen, Feng Ren, Haiyan Zhang, Tao Wen, Zhengfu Piao, Li Zhou, Sujun Zheng, Jing Zhang, Yu Chen, Yuanping Han, Zhongping Duan, Yingji Ma

**Affiliations:** 1 The 2nd Department of Infectious Diseases, the 2nd Affiliated Hospital of Harbin Medical University, Harbin, Heilongjiang Province, People’s Republic of China; 2 Beijing Institute of Liver Diseases, Capital Medical University, Beijing, People’s Republic of China; 3 Beijing Artificial Liver Treatment & Training Center, Beijing Youan Hospital, Capital Medical University, Beijing, People’s Republic of China; 4 The Department of Infectious Diseases, The 4th Affiliated Hospital of Harbin Medical University, Harbin, Heilongjiang Province, People’s Republic of China; Boston University Medical School, United States of America

## Abstract

**Background:**

Glycogen synthase kinase 3β(GSK3β) is a ubiquitous serine-threonine protein kinase that participates in numerous cellular processes and disease pathophysiology. We aimed to determine therapeutic potential of GSK3β inhibition and its mechanism in a well-characterized model of lipopolysaccharide (LPS)-induced model of acute liver failure (ALF).

**Methodology:**

In a murine ALF model induced by D-GalN(700 mg/kg)/LPS(10 µg/kg), we analyzed GSK3β mechanisms using a specific chemical inhibitor, SB216763, and detected the role of endoplasmic reticulum stress (ERS). Mice were administered SB216763 at 2 h before or after D-GalN/LPS injection, respectively, and then sacrificed 6 h after D-GalN/LPS treatment to evaluate its prophylactic and therapeutic function. The lethality rate, liver damage, ERS, cytokine expression, MAP kinase, hepatocyte apoptosis and expression of TLR 4 were evaluated, respectively. Whether the inhibition of GSK3β activation protected hepatocyte from ERS-induced apoptosis was investigated *in vitro*.

**Principal Findings:**

GSK3β became quickly activated (dephosphorylated) upon D-GalN/LPS exposure. Administration of SB216763 not only ameliorated liver injury, as evidenced by reduced transaminase levels, and well-preserved liver architecture, but also decreased lethality. Moreover, GSK3β inhibition resulted in down-regulation of pro-apoptotic proteins C/EBP–homologous protein(CHOP) and caspase-12, which are related to ERS. To further demonstrate the role of ERS, we found that GSK3β inhibition protected hepatocyte from ERS-induced cell death. GSK3β inhibition down-regulated the MAPK pathways, reduced expression of inflammatory cytokines and decreased expression of TLR4.

**Conclusions:**

Our findings demonstrate the key function of GSK3β signaling in the pathophysiology of ALF, especially in regulating the ERS, and provide a rationale for targeting GSK3β as a potential therapeutic strategy to ameliorate ALF.

## Introduction

Acute liver failure (ALF) is a dramatic clinical syndrome that results from massive hepatocyte death. The prognosis of ALF is extremely ominous, and there is no effective therapy for the disease other than liver transplantation [1]. The model of hepatic injury induced by simultaneous injection of D-galactosamine (D-GalN) and lipopolysaccharide (LPS) has been widely used to examine the mechanisms of ALF, which produces typical hepatic apoptosis and necrosis [2]–[4]. Although it is well known that hepatocytes are the major target cells in ALF, and inflammatory cells, such as macrophages and neutrophils, dominate the manifestation of liver injury [5]–[7], the mechanisms of D-GalN/LPS-induced liver injury are not completely understood.

The endoplasmic reticulum (ER) is an organelle that has essential roles in multiple cellular processes that are required for cell survival and normal cellular functions. ER maintains as a cellular sensing system handling variety of stresses ranging from excessive accumulation of proteins, misfolding of proteins, and the sustained loss of Ca^2+^ from lumen of ER. These lead to adaptive responses that dampen the stress or to pathological responses designed to kill the cells themselves that are unable to adequately adapt the stress. The adaptive or protective response is referred to as the unfolded protein response (UPR), while the pathological response is referred to as the ER stress response. The 78-kDa glucose-regulated/binding immunoglobulin protein (GRP78) is a hallmark for both the UPR and ERS responses [8], [9]. The UPR responds rapidly to ERS to enhance cell survival. However, if protein aggregation is persistent and the stress cannot be resolved, signaling switches from pro-survival to pro-apoptotic ERS response. Compelling evidence suggests that c-jun-N-terminal kinase (JNK), C/EBP–homologous protein (CHOP) and the caspase 12 in rodents (caspase 4 in humans) are recruited to participate in ERS-induced apoptosis [10], [11]. Furthermore, a growing body of evidence suggests that the signaling pathways in the UPR and inflammation are interconnected through various mechanisms, including the production of reactive oxygen species (ROS), the release of calcium from the ER, the activation of the transcription factor nuclear factor-κB (NF-κB) and the mitogen-activated protein kinase (MAPK) [12].

GSK3 is a ubiquitously expressed serine/threonine kinase that is initially found to regulate glycogen synthesis. There are two highly homologous isoforms, designated as GSK3α and GSK3β respectively. Constitutively active in resting cells, GSK3β has broad range of substrates and regulates cell activation, differentiation and survival [13], [14]. Among the diverse functions that are regulated by GSK3β, inflammation has recently emerged as one of the major interesting focuses. Studies showed that GSK3 is an important positive regulator of the inflammatory process [15]–[17]. Additionally, GSK3β promotes cell death caused by the intrinsic apoptotic pathway, but inhibits the death receptor-mediated extrinsic apoptotic signaling pathway [18]. GSK3β deletion results in embryonic lethality caused by severe liver degeneration during development [19]. Particularly, GSK3β deficient cells become more sensitive to TNF-α induced apoptosis [20]. Furthermore, GSK3β has been also implicated in ERS-induced apoptosis, which is a new intrinsic apoptotic pathway, and GSK3β oppositely regulates the ERS-induced apoptotic signaling pathway [21]–[23].

The therapeutic potential of GSK3β inhibition needs further confirmation in clinically relevant animal disease models. Although, GSK3β inhibition effectively protected mice and rats from endotoxin shock, its role in acute liver failure induced by D-GalN/LPS needs to be explored, and the underlying mechanisms of its hepatoprotective effects *in vivo* is also needed to be elucidated. Thus, the purposes of this study were: (1) to evaluate the activity of GSK3β and ERS during the progress of ALF, (2) to investigate the role of GSK3β inhibition in protecting liver from lethal injury in response to D-GalN/LPS, and (3) to evaluate GSK3β in the ERS of ALF. Indeed, our results demonstrated a pivotal role of GSK3β in regulating inflammatory process and hepatocyte apoptosis, particularly in the hepatocyte apoptosis induced by ERS in acute liver failure, and revealed the clinical potential of GSK3β inhibition in preventive and therapeutic applications for acute liver failure.

## Materials and Methods

### Animals and Treatment

Male wide-type (WT, C57BL/6) mice (8–12 weeks old) were purchased from the Capital Medical University (Beijing, China ), and housed in the Capital Medical University animal facility under specific pathogen-free conditions, and received humane care according to Capital Medical University Animal Care Committee guidelines. The animal protocol had been approved by the Institutional Animal Care & Use Committee (IACUC) of Capital Medical University. To induce acute liver failure, the mice (except for the control) were injected intraperitoneally with D-GalN (700 mg/kg; Sigma) and LPS (10 µg/kg; Escherichia coli, Sigma) dissolved in phosphate-buffered saline. The inhibitor of GSK3β (SB216763 in DMSO, Sigma) was suspended in PBS and administered intraperitoneally 2 h prior to or after the D-GalN/LPS treatment, respectively. At selected time points after D-GalN/LPS treatment, mice were anesthetized and blood was collected. The liver was harvested and used immediately to prepare mRNA. Both the mRNA and liver tissues were stored at −75°C for later analysis.

### Serum Aminotransferase Activities

Plasma samples were taken from the mice at 6 h after D-GalN/LPS injection. Serum levels of alanine aminotransferase (ALT), aspartate aminotransferase (AST) as markers of hepatic damage were measured by using a multiparameteric analyzer (AU 5400, Olympus, Japan), according to an automated procedure.

### Histopathological Analysis

Liver tissues were fixed in formalin and embedded in paraffin wax, and sections in 5 um were stained with hematoxylin and eosin (H&E) using a standard protocol, and then analyzed by light microscopy. Histological severity of liver injury was graded using Suzuki’s criteria on a scale from 0–4. The liver without necrosis and congestion/centrilobular ballooning was given a score of 0, while severe congestion/degeneration with >60% lobular necrosis is given a value of 4.

### Quantitative Reverse-transcription Polymerase Chain Reaction

Total RNA was isolated from hepatic samples using Trizol reagent according to the manufacturer's protocol. Two and a half ug of RNA was reverse-transcribed into cDNA using SuperScriptTM III First-Strand Synthesis System (Invitrogen, Carlsbad, CA). Quantitative-PCR was performed using the DNA Engine with Chromo 4 Detector (MJ Research, Waltham, MA). In a final reaction volume of 25 µl, the following were added: 1×SuperMix (Platinum SYBR Green qPCR Kit, Invitrogen, Carlsbad, CA), cDNA (2 µl) and 0.5 uM of each primer. Amplification conditions were: 50°C (2 min), 95°C (5 min) followed by 50 cycles of 95°C (15 s), 60°C (30 s). Primers used to amplify a specific mouse gene fragments are listed in Table 1.

**Table 1:** Sequences of the primers for SYBR Green real-time RT-PCR.

Target gene	Forward primers	Reverse primers
HPRT	5-TCAACGGGGGACATAAAAGT-3	5-TGCATTGTTTTACCAGTGTCAA-3
IL-10	5-ACTGCACCCACTTCCCAGT-3	5-TGTCCAGCTGGTCCTTTGTT-3
IL-12p40	5-CAGCTTCTTCATCAGGGACAT-3	5-CTTGAGGGAGAAGTAGGAATGG-3
TNF-α	5-GCCTCTTCTCATTCCTGCTTGT-3	5-TTGAGATCCATGCCGTTG-3
IL-1β	5-TTGACGGACCCCAAAAGAT-3	5-GATGATCTGAGTGTGAGGGTCTG-3
IL-6	5-GCTACCAAACTGGATATAATCAGGA-3	5-CCAGGTAGCTATGGTACTCCAGAA-3
TLR4	5-GCTTTCACCTCTGCCTTCAC-3	5-GAAACTGCCATGTTTGAGCA-3

### Myeloperoxidase Activity Assay

Presence of myeloperoxidase (MPO) was used as an index for neutrophil accumulation in the liver. Briefly, frozen tissue was thawed and weighed, and 100 mg tissue was placed in 4 ml of iced 0.5% hexadecyltrimethyl-ammonium bromide and 50 mM of potassium phosphate buffer solution with the pH adjusted to 5. Each sample was then homogenized for 30 sec and centrifuged at 15,000 rpm for 15 min at 4°C. Supernatants were mixed with hydrogen peroxide–sodium acetate and tetramethylbenzidine solutions. The change in absorbance was measured spectrophotometry at 655 nm. This absorbance was then corrected for the weight of the tissue sample, and results are expressed as specific enzyme activity.

### Determination of Hepatic Caspase-3 Activity

To determine the activity of caspase-3 in the liver tissue of mouse, liver homogenates were made in lysis buffer and analysed using a colorimetric caspase-3 assay kit (Chemicon International Co.) according to the manufacturer’s instruction.

### TUNEL Assay

Apoptosis in liver sections was detected by terminal deoxynucleotidyl transferasemediated dUTP nick-end labeling (TUNEL, red fluorescence) using the In Situ Cell Death Detection Kit (Roche, Indianapolis, IN). Negative control was prepared through omission of terminal transferase. Positive controls were generated by treatment with DNase. Nuclei were stained with 49,6-diamino-2-phenylindole (DAPI, 1 µg/ml) for 10 min. Images were performed on a Nikon Eclipse E800 fluorescent microscope.

### Immunofluorescence Staining

Liver cryosection were fixed by cold methanol followed by permeabilization with 0.1% Triton X100 in PBS and blocked with 10% bovine serum albumin (BSA). Then the sections were incubated with rabbit antibody against active caspase 3 (1∶150, abcam) and PE conjugated anti-mouse F4/80 (1∶150, Biolegend) overnight. To detect active caspase 3, the cells were incubated with goat anti-rabbit IgG-FIFC (1∶200, Santa Cruz Biotech) for 1 hour. Nuclei were stained with DAPI (1 µg/ml) for 10 min. Images were performed on a Nikon Eclipse E800 fluorescent microscope.

### Cell Cultures

Mouse Hepa 1 cells (purchased from Xiangfu Biological Company of Shanghai) were plated in 96 or 48-well plate at appropriate density. After over night culture, tunicamycin (10 µg/ml, Sigma) was added into the culture wells. To study the effect of GSK-3β inhibitors on hepatocyte apoptosis induced by ERS, SB216763 was added 2 h before tunicamycin treatment. Cell death was evaluated at 12 h by western blot for Caspase-3, and 24 h later by LDH assay (Biochain Institute, Hayward, CA) of culture supernatants, according to the manufacturer instruction.

### Western Blot Analysis

Protein was extracted from liver tissue in RIPA buffer together with phosphatase and protease inhibitors. Proteins (30 ug sample) in SDS-loading buffer were subjected to SDS–12% polyacrylamide gel electrophoresis (PAGE) and transferred to PVDF membrane (Bio-Rad, Hercules, CA). Monoclonal rabbit antibodies against p-GSK3β, GSK3β, p-ERK, ERK, p-JNK, JNK, p-P38, P38, GRP78, Caspase-12, Caspase-3, TLR4, β-actin mAb and mouse CHOP mAb (Cell Signaling Technology, Danvers, MA) were used at 4°C overnight. Then the membrane was treated with horseradish peroxidase-conjugated goat anti-rabbit or goat anti-mouse secondary antibodies, and relative quantities of proteins were determined with a densitometer and expressed as absorbance units (AU).

### Statistical Analysis

Results are shown as mean±SD. Statistical analyses were performed using unpaired Student’s t test with p<0.05 (two tailed) considered as significant.

## Results

### The GSK3β Activation/inactivation Profile and ERS in GalN/LPS-induced Acute Liver Failure

**Figure 1:**
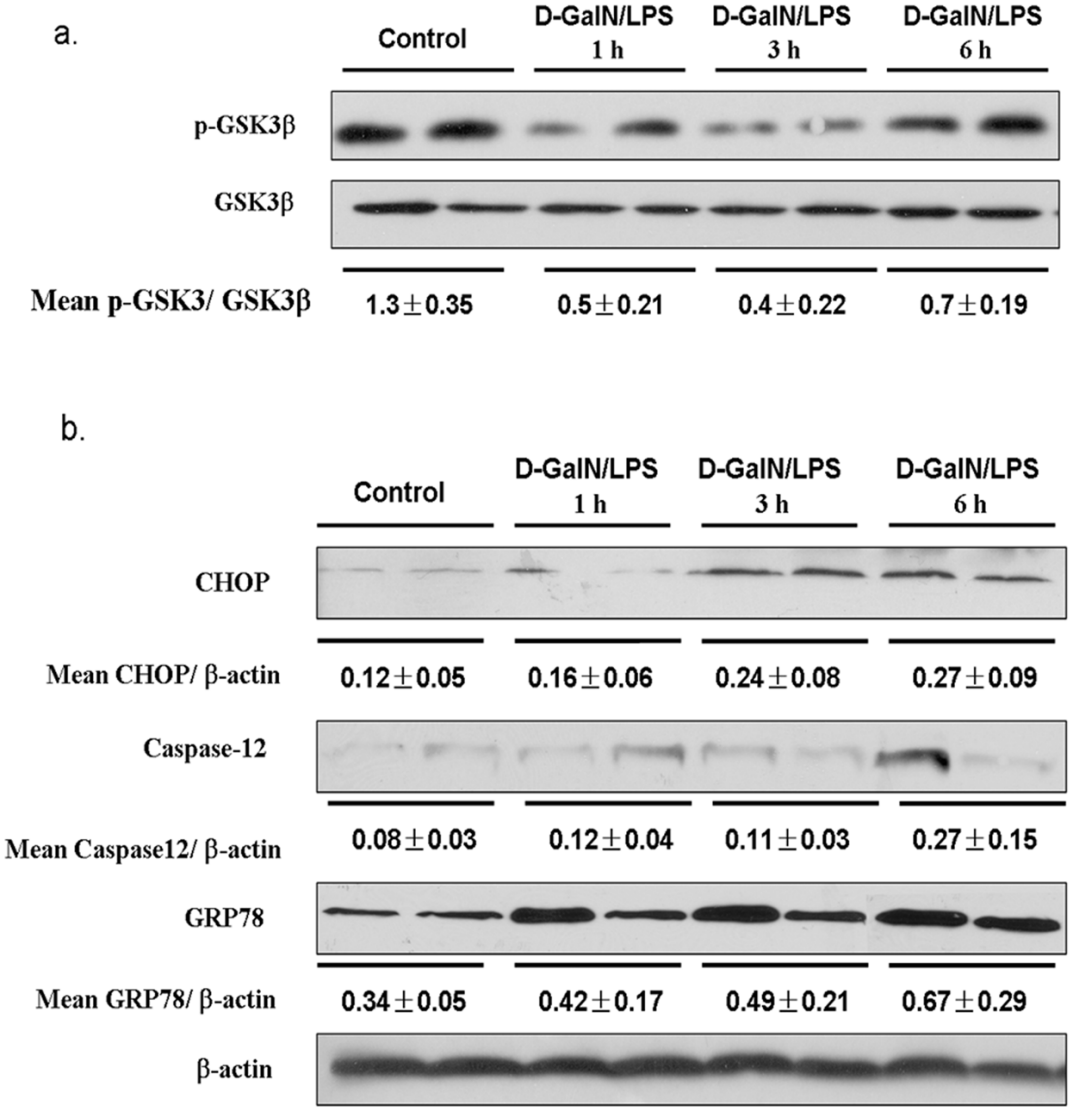
The acute liver failure induced by D-GalN/LPS triggers GSK3β phosphorylation and ERS. Liver samples were harvested from C57BL/6 mice that were subjected to PBS (Control, n = 5–6) or D-GalN/LPS, followed by various times (1, 3, and 6 h, n = 5–6). Representative of two experiments is shown. Densitometry analysis of the proteins was performed for each sample (mean±SEM). (a)The phosphorylated (serine 9) and total GSK3β levels were measured by Western blots. (b)The ERS-related protein including GRP78, CHOP, Caspase-12, and β-actin were measured by Western blots.

To dissect the role of GSK3β in the pathogenesis of liver failure, we first determined *in vivo* whether the GSK3β phosphorylation/dephosphorylation is triggered in acute liver injury. Liver tissues were harvested in 1, 3, and 6 h, respectively, after D-GalN/LPS injection. Compared with those in the negative control, the phosphorylated (serine 9) GSK3β level in the liver tissues was promptly reduced in 1 h and 3 h, and then was restored after 6 h, suggesting dephosphorylation of GSK3β is an early event in the acute phase (Fig.1a). As total GSK3β levels were unchanged among the different time points, our result indicates that the liver GSK3β activity, rather than its protein, was increased during early phase of liver failure induced by D-GalN/LPS, but declined to the basal state thereafter. On the other hand, phosphorylation at tyrosine-216 on GSK3β and the phosphorylation of GSK3α were not detected in the acute liver failure (data not shown). Thus, the GSK3β activation, as measured by dephosphoryaltion at serine-9, is triggered in the early phase of the D-GalN/LPS induced liver failure.

To evaluate the role of ERS in acute liver failure induced by D-GalN/LPS, expression of GRP78, CHOP, and caspase-12 in the liver of D-GalN/LPS-treated mice was determined by Western blot analysis. The expression of GRP78, CHOP, caspase-12, was low in the control liver, but all increased significantly from 3 to 6 h after D-GalN/LPS administration (Fig. 1b). The increased expression of GRP78, CHOP and caspase-12 suggested that endoplasmic reticulum stress took place in a delayed fashion in the D-GalN/LPS-induced acute liver failure. These results suggest that ERS may play an important role in D-GalN/LPS-induced acute liver failure.

### Active GSK3β is Critical for the Development of ALF Induced by D-GalN/LPS

**Figure 2:**
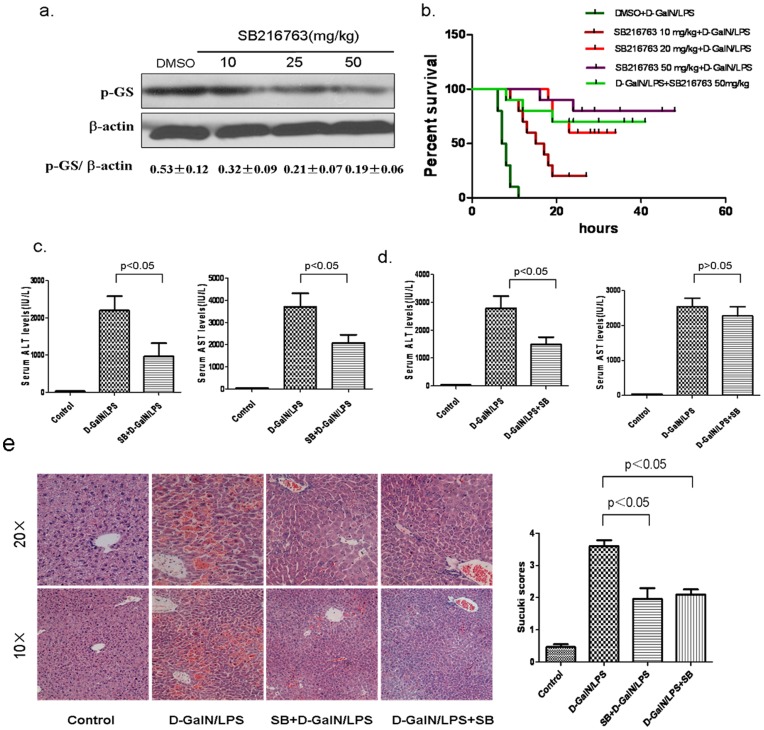
The GSK3β inhibition by SB216763 ameliorates liver injury induced by D-GalN/LPS in C57BL/6 mice. (a) Liver samples, harvested 6 h later, were subjected to Western blot analysis of phosphorylated glycogen synthases. Wild-type mice were pretreated with vehicle (DMSO, n = 3) or SB216763 (10, 25, 50 mg/kg, respectively, n = 3). Representative of one experiment is shown. Densitometry analysis of the proteins was performed for each sample (mean±SEM). (b)GSK3β inhibition enhances the survival rate of mice after D-GalN/LPS injection. SB216763 (10, 25, or 50 mg/kg body weight) or vehicle (DMSO) was intraperitoneally administered at 2 h before D-GalN/LPS injection (DMSO+D-GalN/LPS, SB216763 10 mg/kg+D-GalN/LPS, SB216763 25 mg/kg+D-GalN/LPS, SB216763 50 mg/kg+D-GalN/LPS); and the fifth group is administered SB216763 at 2 hour after D-GalN/LPS(D-GalN/LPS+SB216763 50 mg/kg). (n = 10/group). (c) Wild-type mice (n = 8–12) pretreated SB216763 before D-GalN/LPS injection were analyzed for serum ALT and AST level at 6 h after D-GalN/LPS. (d)Wild-type mice (n = 8–12) treated SB216763 after D-GalN/LPS injection were analyzed for serum ALT and AST level at 6 h after D-GalN/LPS. (e) Representative liver histology (H/E staining at 6 h after D-GalN/LPS) and the group averages of liver Suzuki scores (6 h). Control: DMSO groups, DMSO+D-GalN/LPS: model group, SB+D-GalN/LPS: prophylactic group and D-GalN/LPS+SB: therapeutic group. (n = 5–6/group).

To address the functional significance of GSK3β in liver injury, we treated mice with a GSK3β specific chemical inhibitor, SB216763, 2 hours prior to or after the onset of liver failure. The inhibition of liver GSK3 activity *in vivo* was confirmed by reduced phosphorylation of glycogen synthase, a downstream substrate of GSK3β (Fig.2a).

For mortality analysis, four treatment groups, all challenged with D-GalN/LPS, were examined. The mice in group I (control) received only vehicle, and the mice in groups II to IV were pretreated with three doses of SB216763 before D-GalN/LPS challenge; and the mice in group V were administered SB216763 in 2 hours after onset of ALF by D-GalN/LPS. The survived mice of all groups were sacrificed at 48 h. In the D-GalN/LPS control group, the mice began to die 6 h after D-GalN/LPS injection, and all mice were dead in 20 h after D-GalN/LPS injection (mortality 100%). However, pretreatment with SB216763 reduced the lethality in a dose-dependent manner. Briefly, in 48 h after D-GalN/LPS administration, the lethality rates were 80%, 60%, and 20% (survival of 2, 4, and 8 of 10 mice) in the mice pretreated with 10, 25, and 50 mg/kg SB216763, respectively; For the therapeutic treatment of SB216763(50 mg/kg), the lethality rate was 40% (survival of 6 of 10 mice), as showed in Fig. 2b. SB216763 (50 mg/kg) showed significantly protection from liver failure. Therefore, we selected 50 mg/kg of SB216763 for the following study.

To address the effect of inhibiting activity of GSK3β on hepatic injury, we investigated whether SB216763 regulates serum AST and ALT levels in D-GalN/LPS-induced liver failure. After 6 h of D-GalN/LPS-induced liver failure, sALT and sAST levels reached 2195.3±450.5 and 3705.3±632.8 IU/L, which were significantly higher than those in single SB216763 treated ones (961.1±356 and 2709.9±423.9), while vehicle-treated normal mice levels were 28.7±7 and 33.7±9 IU/L, respectively (Fig. 2c ). For therapeutic function of SB216763, as compared with D-GalN/LPS-treated positive groups, livers in animals receiving SB216763 also suffered less acute liver failure injury, evidenced by distinct lower sALT levels (Fig. 2d, sALT:1491.2±622.9 versus 2787.52±1066.89; P<0.05), but the sAST level had no statistics difference (2274.5±651 versus 2538.13±588.9; P>0.05). Liver histology was normal in the vehicle-treated normal mice, while hepatic architecture in 6 h after D-GalN/LPS administration was disrupted with the appearance of extensive areas of hemorrhage and coagulative necrosis. However, little hemorrhage and no evidence of necrosis were observed in livers from SB216763 treated mice at this time, better preserved liver architecture by histology and Suzuki grading (Fig. 2e, 4.1±0.2, 3.5±0.6 and 3.6±0.8, P<0.05). Taken together, these results demonstrate not only the prophylactic, but also the therapeutic application of SB216763, being used an agent to protect the liver from the D-GalN/LPS-induced ALF in the mice.

**Figure 3:**
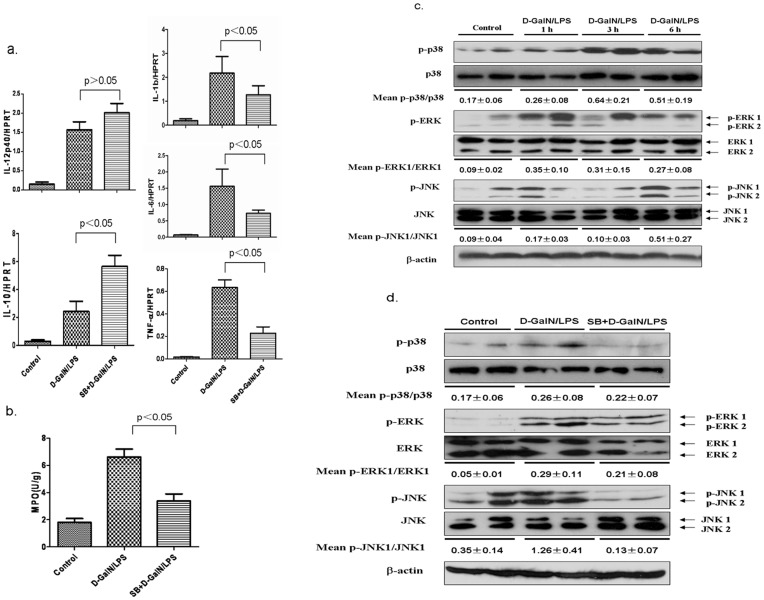
Liver protection against acute liver failure following GSK3β inhibition is dependent of regulating inflammatory reaction and modulating the MAP kinases signaling. (a) The gene expression levels of inflammatory IL-12p40, IL-10 at 2 hour after D-GalN/LPS injection, and TNF-α, IL-1β and IL-6 at 6 h after D-GalN/LPS injection(n = 5–6/group). (b) Liver MPO levels at 6 h after D-GalN/LPS injection (n = 5–6/group). (c) The phosphorylated MAP kinases including JNK, ERK and p38, and β-actin were measured by Western blots. Liver samples were harvested from C57BL/6 mice that were subjected to PBS (Control, n = 5–6) or D-GalN/LPS, followed by various times (1, 3, and 6 h, n = 5–6/group). Representative of two experiments is shown. Densitometry analysis of the proteins was performed for each sample (mean±SEM). (d) The MAP kinases are regulated by GSK3β inhibition in SB216763-pretreated acute liver failure in mice. Groups of C57BL/6 mice were treated with vehicle (DMSO, n = 5–6/group), or SB216763 (50 mg/kg, n = 5–6/group), followed by 6 h D-GalN/LPS injection. Representative of two experiments is shown. Densitometry analysis of the proteins was performed for each sample (mean±SEM).

### Active GSK3β Facilitates Cytokine Programs, Neutrophil Infiltration and Regulates the MAPK Pathways

LPS can trigger inflammatory cascades involving the induction of pro-inflammatory cytokines including TNF-α, IL-1β and IL-6, which are essential for inflammation and consequent liver damage in D-GalN/LPS-treated mice. In order to determine the impact of GSK3β inhibition on the induction of these cytokines by ALF, the livers were harvested at 4 h after D-GalN/LPS injection. Indeed, inhibition of GSK3β attenuated the expression of pro-inflammatory cytokines including TNF-α, IL-1β, and IL-6, and conversely increased immune regulatory cytokines such as IL-12 and IL-10 in the acute phase (Fig.3a). These results also suggest a positive role of GSK3β in regulation of pro-inflammatory responses. To investigate D-GalN/LPS-induced neutrophil infiltration, we assayed MPO activities. As shown in Fig.3b, treatment with SB216763 significantly decreased the injury related MPO activities (3.94±0.58), as compared with the D-GalN/LPS control (6.425±1.24, p<0.05). Meanwhile, we analyzed to which extent macrophages are involved in the inflammatory response of the D-GalN/LPS exposed livers by immunofluorescence staining to detect the PE anti-mouse F4/80. The results showed that there are no difference among all groups (data not shown). Thus, inhibition of GSK3β activity suppressed expression of pro-inflammatory cytokine, and reduced neutrophil activation in D-GalN/LPS induced liver failure.

**Figure 4:**
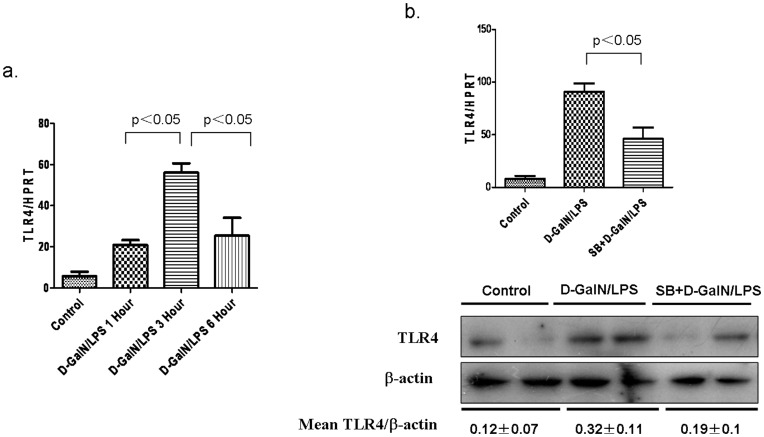
Active GSK3β promotes TLR4 expression in D-GalN/LPS-induced ALF. (a) Liver samples were harvested from C57BL/6 mice that were subjected to D-GalN/LPS (Control, 1 h, 3 h and 6 h, n = 5–6/group), the gene expression levels of TLR4 after D-GalN/LPS injection were tested by qRT-PCR. (b) Liver samples were harvested from C57BL/6 mice at 6 h after D-GalN/LPS injection that were pretreated with only PBS (Control, n = 5–6), and vehicle (DMSO, n = 5–6/group), or SB216763 (50 mg/kg, n = 5–6/group) before D-GalN/LPS injection. Hepatic gene and proteins expression of TLR4 were measured by Western blots and qRT-PCR, respectively. For western blot analysis, representative of two experiments is shown. Densitometry analysis of the proteins was performed for each sample (mean±SEM).

We then assessed the downstream events of the pro-inflammatory cytokine receptors, namely activation of MAP kinases including ERK, JNK, and p38. As shown in Fig. 3c, these three MAP kinases in livers showed different pattern of activation dynamics. Phosphorylation of ERK and p38 was significantly increased in 1 h and maintained at a significantly high level for 3 h after D-GalN/LPS injection, while phosphorylation of JNK proteins was apparently in oscillation with a prompt increase after 1 h, followed by a down turn, and another momentum at 6 h. These results suggest that the MAP kinases which involved in diverse functions were differently regulated in D-GalN/LPS induced ALF. Next, we examined whether the MAPK pathways were involved in the protective effect in D-GalN/LPS–induced ALF by inhibition of GSK3β. As shown in Fig. 3d, compared with the control, the pretreatment with SB216763 decreased the levels of phospho-ERK and phospho-p38 MAPK in 3 h and the level of phospho-JNK in 6 h after D-GalN/LPS treatment. These results indicated that the GSK3β may act upstream or modulate the inflammation related three MAPK pathways.

**Figure 5:**
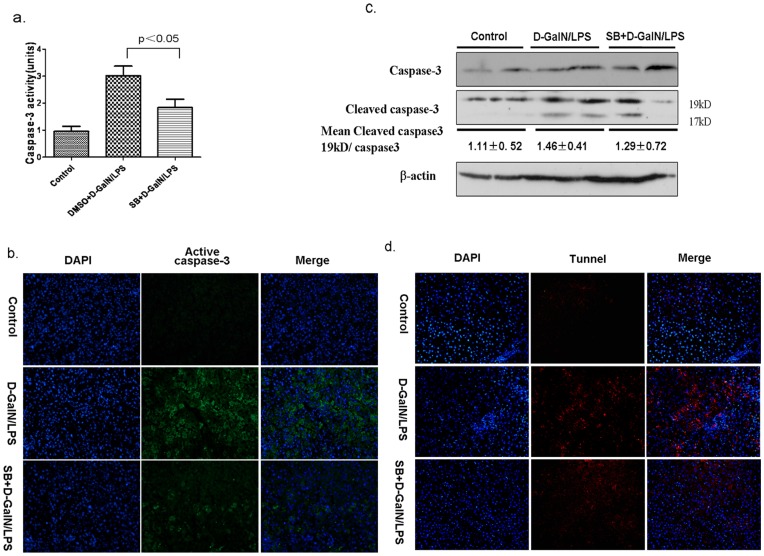
The Inhibition of GSK3β prevents hepatocyte apoptosis in D-GalN/LPS-induced acute liver failure. Liver samples were harvested from C57BL/6 mice at 6 h after D-GalN/LPS injection that were pretreated with only PBS (Control, n = 5–6), and vehicle (DMSO, n = 5–6/group), or SB216763 (50 mg/kg, n = 5–6/group) before D-GalN/LPS injection. (a)Caspase-3 activity assay at 6 h after D-GalN/LPS injection, controls were defined as 1.0. (b)Immunofluorescence staining for active caspase-3 (green) in the liver of mice. Representative of one experiment is shown. Original magnification×20. (c)Hepatic proteins expression of caspas-3, cleaved caspase-3 and β-actin were measured by Western blots. Representative of two experiments is shown. Densitometry analysis of the proteins was performed for each sample (mean±SEM). (d) TUNEL staining (red) liver tissue at 6 h after D-GalN/LPS administration. Representative of one experiment is shown. Original magnification×20.

### Active GSK3β Promotes TLR4 Expression in Liver

Toll-like receptors (TLRs) regulate systemic acquired immunity and local infammatory responses, the studies have revealed that TLRs involve in the initiation, progression and recovery of various liver diseases. At here, our results showed that the expression of TLR4 mRNA evidently increased at the 3 hours and decreased at 6 hours after D-GalN/LPS stimulation (Fig.4a). The inhibition of GSK3β decreased the expression of TLR4 mRNA and TLR4 proteins at 3 hours detected by qRT-PCR and western blot analysis, respectively(showed as Fig.4b and 4c). So, the active GSK3β signaling promotes the TLR4 status in D-GalN/LPS-induced ALF.

**Figure 6:**
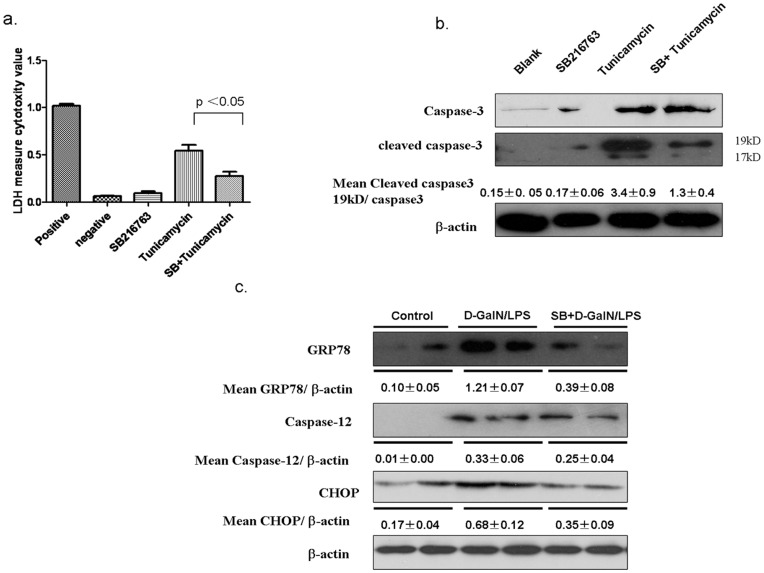
The Inhibition of GSK3β prevents ERS-induced hepatocyte apoptosis *in vitro* and *in vivo*. (a) LDH activities assay in the hepatocyte culture supernatant. SB216763 was added 2 h before tunicamycin(10 µg/ml, Sigma) which is added into the 24 wells culture plate for 24 hours(n = 3 wells). (b) The hepatocyte proteins expression of caspas-3, cleaved caspase-3 and β-actin were measured by Western blots. SB216763 was added 2 h before tunicamycin(10 µg/ml, Sigma) which was added into the 60 cm culture well for 12 hours, total lysates were subjected to immunoblot assay, and densitometry analysis(mean±SEM) was performed (n = 3). (c) The liver proteins expression of ERS (GRP78, CHOP, caspase-12) were measured by Western blots. Liver samples were harvested from C57BL/6 mice at 6 h after D-GalN/LPS injection that were pretreated with only PBS (Control, n = 5–6), and vehicle (DMSO, n = 5–6/group), or SB216763 (50 mg/kg, n = 5–6/group) before D-GalN/LPS injection. Representative of two experiments is shown. Densitometry analysis of the proteins was performed for each sample (mean±SEM).

### GSK3β Signaling Promotes Hepatocyte Apoptosis during ALF

As D-GalN/LPS-induced liver injury is characterized by apoptosis of hepatocytes, the expression and activity of pro-apoptotic molecules were examined. In animal model, capase-3 activity, active caspase-3 and its maturation/process of the proenzyme was markedly increased in D-GalN/LPS induced liver failure (Fig. 5a, 5b and 5c). Treatment with SB216763 significantly inhibited the caspase-3 activity, active caspase-3 and the cleavage of procaspase-3. Similar results were also observed in liver tissue samples by applying TUNEL assay (Fig. 5d). Therefore, GSK3β inhibition was capable of inhibiting hepatocyte apoptosis in hepatic failure induced by D-GalN/LPS.

### Inhibition of GSK3β Prevents ERS-induced Hepatocyte Apoptosis

As showed above, ERS play an important role in the mechanism of D-GalN/LPS-induced ALF, so we investigated the role of GSK3β in the intrinsic potential of hepatocyte death triggered by ERS pathway. First, we determined the impact of GSK3β inhibition on ERS-induced mouse Hepa 1 cell death *in vitro*. GSK3β inhibition did protect hepatocytes from tunicamycin-induced ERS and cell death, as measured by the LDH assay and Western blotting for cleaved caspase-3. There was significant decrease in LDH activities in the hepatocyte culture supernatant by the cells pretreated with SB216763, as compared with controls, incubated with tunicamycin (Fig.6a). Western blot analysis showed that SB316763 inhibited the expression of cleaved caspase-3 compared with controls (Fig. 6b). By *in vivo* animal model, Western blot analysis revealed that treatment with SB216763 inhibited the expression of CHOP and caspase-12 compared with controls in 6 hours (Fig. 6c). Therefore, GSK3β inhibition was capable of inhibiting hepatocyte death against ER stress.

## Discussion

GSK3β has been shown to regulate macrophage cytokine production and hepatocyte apoptosis [24], [25], however, its role in acute liver failure–the inflammation and apoptosis-mediated hepatocellular injury process–has not been explored. By *in vivo* experiments, GSK3β inhibition has been shown to protect mice from endotoxin induced shock [15]. Compared with the effects of LPS alone, the administration of LPS together with or a sub-lethal dose of D-galactosamine (D-GalN) induced more severe hepatic damage accompanied by apoptotic and necrotic changes in the liver, which is similar to human liver failure [26], [27]. Therefore, it is of interest to further investigate the hepatoprotective potential and the mechanism of GSK3β inhibition in D-GalN/LPS induced ALF. Our current study demonstrates (1) that GSK3β is activated in the acute phase of ALF, and inhibition GSK3β can ameliorate the toxin induced liver failure, suggesting its clinical potentials, (2) that the role of GSK3β in ALF may act upstream or modulate the major MAPK pathways and by induction of pro-inflammatory cytokines, (3) that GSK3β may either act modulate TLR4 expression in amplification of injury signals, and (4) that activation GSK3β may control ERS and its dependent apoptosis in ALF.

Our study demonstrates GSK3β in controlling inflammation by two lines of evidence. First, inhibition GSK3β suppresses the expression of pro-inflammatory cytokines, indicating that the kinase may positively participates in expression of inflammatory cytokine gens in the liver injury. Second, inhibition of GSK3β activity promotes IL-10 and IL-12 transcription, indicating that the kinase may negatively suppresses the immune regulatory cytokines in liver damage. GSK3β has been linked to the regulation of a multitude of transcription factors, including NF-κB, AP-1, NF-AT and CREB [15]. Inhibiting GSK3β allows CREB to sequester CBP from NF-κB, promoting CREB-dependent IL-10 production and suppressing NF-κB-dependent inflammatory cytokine expression. Our previous study also showed that the inhibition of GSK3β ameliorates liver ischemia reperfusion injury via an IL-10-mediated immune modulatory mechanism [28]. The current study reveals critical roles of GSK3β in acute liver failure through defined down-stream pathways.

In healthy tissues, GSK3β is retained in an inactivated form by phosphorylation at serine-9 through upstream AKT which is activated by survival signals including growth factors (EGF, IGF) and WNT ligands [29]. Under injury signals such as TNF-alpha and IL-1, four MAP kinase pathways, including ERK, JNK, and p38 MAPK, are activated to fulfill their acute response by expression inflammatory effectors or mediators such as MMPs. Here we showed that inhibition of GSK3β attenuates the MAPK activation and the down-stream cytokine transcription in toxin-induced ALF. Such notion was also demonstrated by a recent report showing GSK3β in mediating MAPK activation and cytokine production by mast cells [30]. JNK activation is an important component of the stress response in cells, but when JNK activation is sustained, it is believed to promote cell injury and death. In this study, the enhanced activation of MAPK in D-GalN/LPS induced ALF was observed in comparison to the control. And moreover, SB216763 pretreatment significantly inhibited D-GalN/LPS–induced activation of JNK, ERK and p38. At molecular level, it is still not clear how GSK3β activate MAPK. Our previous report showed that GSK3β inhibition did not alter LPS-induced MAPKs activation in macrophage cultures [28], but others using mast cells demonstrated inhibition GSK3β can attenuate JNK activation. How GSK3β regulates MAPK in liver injury will be further studied in primary hepatic cells.

Toll-like receptors (TLRs) can recognize pathogen associated molecular patterns (PAMPs) to participate in the immune response, and TLR4 is the recognition receptors of LPS which involved in microbial immune response and cell activation. In acute liver failure, the expression of TLR4 of hepatic cell and its effects is still some controversy. Some results show that TLR4 expression was decreased in acute liver failure [31]; another study showed that TLR4 expression was significantly increased with the liver injury [32]. In this study, we further investigate expression of TLR4 in D-GalN/LPS response and its role in protective effect of GSK3β inhibition. Our results show that, with the progress of ALF, the gene of TLR4 expression was high expression at 3 hours, but low expression at 6 hours, which may be associated with a large number of hepatocyte necrosis in the late phase of ALF. GSK3β inhibition significantly reduced the TLR4 gene and protein expression, as a results, it reduced the sensitivity of liver cells (including macrophages and hepatocytes) to LPS, and led to the improvement of liver injury. So, it may be one of the mechanisms of GSK3β inhibition to improve the D-GalN/LPS-induced ALF.

There are three ways to induce cell apoptosis: death receptor-mediated apoptosis are extrinsic apoptotic pathways, mitochondria and endoplasmic reticulum-mediated apoptosis are endogenous apoptotic pathways [33], [34]. Many studies have shown that GSK3β plays an important role in the regulation of apoptosis, and inhibition of GSK3β not only promote cell apoptosis induced by extrinsic signaling pathways, but also inhibit the cell apoptosis induced by the endogenous signaling pathways [18]. GSK-3β plays an important role in the ERS-induced apoptosis pathway. Certain drugs can induce the ERS, including thapsigargin (ER Ca-ATPase inhibitor) and tunicamycin (N-glycosylation inhibitors). Previous *in vitro* study showed that the GSK-3β inhibitors can reduce ERS-induced apoptosis, and 2-propylamyl sodium can inhibit ERS-induced apoptosis through inhibiting the activity of GSK-3β [23]. The apoptosis induced by thapsigargin and tunicamycin in mouse pancreatic tumor cells can be attenuated by siRNA knocking down GSK-3β [21]. Those GSK-3β inhibitors also significantly ameliorate thapsigargin-induced apoptosis in PC12 cells and HepG2 cell [22]. Our findings suggest that inhibition of GSK3β activity can protect the liver from toxin-induced injury in mice through reduction of parenchymal apoptosis. In this study, we found the expressions of GRP78, CHOP and caspase-12 is up-regulated with the progress of D-GalN/LPS-induced ALF, demonstrating potential role of ERS-mediated hepatocyte apoptosis in ALF. Then we explored the linkage from GSK3β to the ERS-mediated hepatocyte apoptosis: *in vitro*, GSK3β inhibition alleviate hepatocyte apoptosis induced by tunicamycin, and *in vivo,* decreased the expression of CHOP and caspase-12 in liver tissue, which were markers of ERS and participate in the ERS-induced cell apoptosis. Although our results is some different from the study from Nakayama et al which have reported that LPS-induced CHOP expression does not induce apoptosis when macrophages are treated with LPS, while pro-IL-1β is activated [35]. we thinks it is because that the need of D-GalN along with LPS to induce apoptosis clearly demonstrated two hit theory, by which D-GalN created ER stress and over-consumption of UTP failed to induce the anti-apoptotic inhibitor, allowing LPS induced activation of caspase pathway [36].

In summary, GSK3β inhibition represents a potent and safe strategy to ameliorate liver ALF pathology. This approach may provide not only the direct cytoprotection means against ERS-induced cell death, but also exert immune modulation to reduce local inflammation. Further preclinical studies with GSK3β chemical inhibitors are warranted to pave the way for the development of a clinically applicable therapeutic strategy against ALF.
